# Variant rs10911021 that associates with coronary heart disease in type 2 diabetes, is associated with lower concentrations of circulating HDL cholesterol and large HDL particles but not with amino acids

**DOI:** 10.1186/s12933-016-0435-0

**Published:** 2016-08-22

**Authors:** Katherine E. Beaney, Jackie A. Cooper, Stela McLachlan, S. Goya Wannamethee, Barbara J. Jefferis, Peter Whincup, Yoav Ben-Shlomo, Jacqueline F. Price, Meena Kumari, Andrew Wong, Ken Ong, Rebecca Hardy, Diana Kuh, Mika Kivimaki, Antti J. Kangas, Pasi Soininen, Mika Ala-Korpela, Fotios Drenos, Steve E. Humphries, on behalf of the UCLEB consortium

**Affiliations:** 1 Centre for Cardiovascular Genetics, British Heart Foundation Laboratories, Institute of Cardiovascular Science, University College London, University Street, London, UK; 2 Centre for Population Health Sciences, The University of Edinburgh, Edinburgh, UK; 3 UCL Department of Primary Care & Population Health, UCL Institute of Epidemiology, University College London, London, UK; 4 Population Health Research Institute, St George’s University of London, Cranmer Terrace, London, UK; 5 School of Social and Community Medicine, University of Bristol, Bristol, UK; 6 Institute for Social and Economic Research, University of Essex, Colchester, UK; 7 Department of Epidemiology & Public Health, UCL Institute of Epidemiology & Health Care, University College London, London, UK; 8 MRC Unit for Lifelong Health and Ageing, London, UK; 9 MRC Epidemiology Unit, Institute of Metabolic Science, Addenbrooke’s Hospital, Cambridge, UK; 10 Computational Medicine, Faculty of Medicine, University of Oulu and Biocenter Oulu, Oulu, Finland; 11 NMR Metabolomics Laboratory, School of Pharmacy, University of Eastern Finland, Kuopio, Finland; 12 MRC Integrative Epidemiology Unit, School of Social and Community Medicine, University of Bristol, Bristol, UK

**Keywords:** Coronary heart disease, Metabolomics, HDL-cholesterol, Genetic risk

## Abstract

**Aims:**

An intergenic locus on chromosome 1 (lead SNP rs10911021) was previously associated with coronary heart disease (CHD) in type 2 diabetes (T2D). Using data from the UCLEB consortium we investigated the relationship between rs10911021 and CHD in T2D, whether rs10911021 was associated with levels of amino acids involved in the γ-glutamyl cycle or any conventional risk factors (CRFs) for CHD in the T2D participants.

**Methods:**

Four UCLEB studies (n = 6531) had rs10911021 imputation, CHD in T2D, CRF and metabolomics data determined using a nuclear magnetic resonance based platform.

**Results:**

The expected direction of effect between rs10911021 and CHD in T2D was observed (1377 no CHD/160 CHD; minor allele OR 0.80, 95 % CI 0.60–1.06) although this was not statistically significant (p = 0.13). No association between rs10911021 and CHD was seen in non-T2D participants (11218 no CHD/1274 CHD; minor allele OR 1.00 95 % CIs 0.92–1.10). In T2D participants, while no associations were observed between rs10911021 and the nine amino acids measured, rs10911021 was associated with HDL-cholesterol (p = 0.0005) but the minor “protective” allele was associated with lower levels (−0.034 mmol/l per allele). Focusing more closely on the HDL-cholesterol subclasses measured, we observed that rs10911021 was associated with six large HDL particle measures in T2D (all p < 0.001). No significant associations were seen in non-T2D subjects.

**Conclusions:**

Our findings are consistent with a true association between rs10911021 and CHD in T2D. The protective minor allele was associated with lower HDL-cholesterol and reductions in HDL particle traits. Our results indicate a complex relationship between rs10911021 and CHD in T2D.

**Electronic supplementary material:**

The online version of this article (doi:10.1186/s12933-016-0435-0) contains supplementary material, which is available to authorized users.

## Background

Data from observational studies has long shown that those with type 2 diabetes (T2D) are at an increased risk of developing coronary heart disease (CHD) [1]. Evidence from genetic studies suggests that this relationship is causal [2, 3]. As such, there is growing interest in factors which may promote a “pro-atherogenic” environment in diabetes. While the association of a number of genetic risk factors for CHD [4] and subclinical cardiovascular disease (CVD) [5] has been observed in diabetic populations, recently a risk locus not previously identified in the general population was found to be associated with CHD in T2D. This locus at chromosome 1q25, (lead SNP rs10911021), was found to be associated with CHD in diabetic individuals [6] (MAF = 0.29 in the CEU group of 1000 Genomes Phase 3). The minor allele had a protective effect and an OR of 0.74 (95 % CI 0.66–0.82, 1517 CHD cases, 2671 controls). The authors also observed that the risk homozygote genotype of rs10911021 was associated with 32 % lower expression of the nearest downstream gene *GLUL* compared to the protective allele homozygote genotype in endothelial cells. *GLUL* encodes the enzyme glutamine-synthase, an enzyme which catalyses the conversion of glutamic acid to glutamine. Furthermore, while no association between levels of glutamic acid or glutamine and rs10911021 was observed, an association between the SNP and the ratio of pyroglutamic acid to glutamic acid was reported. Both metabolites are intermediates in the γ-glutamyl cycle. This cycle is involved in amino acid uptake and in the homeostasis of the anti-oxidant glutathione [7]. Thus, the authors hypothesised that the presence of the risk allele may result in a lesser availability of glutathione. Intracellular glutathione is known to be lower in diabetic individuals [8].

The risk locus identified for CHD in T2D also falls close to a GWAS hit for high density lipoprotein cholesterol (HDL-C) levels, (lead SNP rs1689800) situated between the genes *ZNF648* and *LINC01344* [9]. However, the degree of linkage disequilibrium (LD) between the two lead SNPs was low (r^2^ = 0.03 and D’ = 0.22, calculated from the CEU group of 1000 Genomes pilot). While the minor allele of rs1689800 is associated with 0.01 mmol/l lower HDL-C in the general population, data from the Global Lipids Genetics Consortium did not identify an association between rs10911021 and HDL-C levels (p = 0.50) in the general population [10].

In this study we sought to confirm the reported association between rs10911021 and CHD in T2D, and then to assess if this SNP was associated with amino acid levels as measured using a high-throughput nuclear magnetic resonance (NMR) metabolomics platform. Finally, we sought to assess whether rs10911021 was associated with any conventional risk factors (CRFs) for CHD in the diabetic state, including levels of HDL-C and related HDL particle traits as measured using the high-throughput NMR metabolomics platform.

## Methods

### UCLEB

The University College, London School of Hygiene and Tropical Medicine, Edinburgh and Bristol (UCLEB) Consortium comprises 12 prospective studies, almost all participants of which are of white/European ethnicity. The consortium has been described in detail elsewhere [11]. Median follow-up was 10 years. Approximately 21,000 participants included in the UCLEB studies were genotyped using the Metabochip. This platform has approximately 200,000 SNPs, designed to cover regions associated with cardio-metabolic disease. Imputation based on data from the 1000 Genomes European Ancestry sample extended the SNP coverage to approximately one million SNPs (R^2^ ≥ 0.8), including rs10911021 (R^2^ = 0.95). CHD was defined as the occurrence of fatal CHD, non-fatal myocardial infarction or undergoing coronary artery bypass or angioplasty. Both rs10911021 imputation and CHD outcome data were available for eight cohorts—British Regional Heart Study (BRHS), British Women’s Heart and Health Study (BWHHS), Caerphilly Prospective Study (CAPS), Edinburgh Artery Study (EAS), Edinburgh Type 2 Diabetes Study (ET2DS), English Longitudinal Study of Aging (ELSA), MRC National Survey of Health and Development (MRC1946) and Whitehall II (WHII). Metabolomics and rs10911021 imputation data were available for four studies (BWHHS, ET2DS, MRC1946 and WHII). T2D was defined by self-report, medical history review, taking glucose lowering medication or a fasting glucose >7 mmol/l. The T2D group included only those with prevalent diabetes (either self-reported or clinically confirmed as described in [11]). Informed consent was obtained for all subjects included in UCLEB research. Written approval from individual Research Ethics Committees to use anonymised individual level data have been obtained by each participating study.

### Metabolomics

Serum metabolic measures were quantified using a high-throughput NMR metabolomics platform able to quantify up to 233 metabolic measures representing a broad molecular signature of systemic metabolism [12, 13]. Multiple metabolic pathways are covered, including lipoprotein lipids and lipid subclasses, fatty acids and fatty acid compositions, as well as amino acids and glycolysis precursors. Applications of this high-throughput metabolomics platform have recently been reviewed [12] and details of the experimentation have been described elsewhere [13, 14]. Fasting concentrations of nine amino acid measures and 53 HDL related traits were determined in all four studies with available genotyping/imputation and diabetes status data.

### Statistical analysis

Calculations were performed to assess the power required to detect the effect found by Qi et al. [6] using the QUANTO software package [15]. From the UCLEB data available, three of the studies had a nested case–control design (BRHS, BWHSS and ELSA) while a fourth did not record the times of CHD events (WHII). Therefore, the relationship between rs10911021 and CHD in T2D in UCLEB was assessed using logistic regression adjusted for sex. All participants used in the CHD analysis were free of CHD at baseline. Statistical analysis for the UCLEB metabolomics was performed using R version 3.2.1 [16]. All other analysis was performed using STATA [17]. Meta-analyses was performed using the R package “metafor” using either a fixed effects or random effects (DerSimonian Laird) model [18]. All metabolomics measures were adjusted for age, age^2^ and sex and an inverse rank transformation was used prior to association analysis [19]. This was carried out using a linear model, adjusted for lipid lowering medication use, in each cohort individually. Separate analysis was performed for those with and without prevalent T2D. The results from the different studies were then combined in a fixed-effects meta-analysis weighted by sample size. To account for multiple testing and the correlation between the metabolomic traits p values were adjusted using the false discovery rate (FDR) from Benjamini-Hochberg-Yekutieli [20]. An FDR adjusted *p* value <0.05 was considered to be statistically significant. Conditional analysis with the nearby HDL GWAS hit was performed by including the lead SNP (rs1689800) in the linear model. To assess the number of independent effects observed we performed step-wise adjustment of the results whereby all of the statistically significant associations were re-tested with the metabolomics measure with the lowest p value used a covariate. Should measures remain statistically significant, the process is repeated until none remain.

## Results

### Basic characteristics of UCLEB participants

Selected characteristic of the UCLEB participants, separated by diabetes status, are shown in Table 1. Compared to the non-T2D study population, the diabetes study population (which was heavily influenced by a single study comprising just older people T2D, the ET2DS, n = 1066) had a higher BMI, higher triglycerides, higher blood pressure, fasting glucose, insulin and glycated haemoglobin. Conversely, the non-T2D participants had higher total cholesterol and higher HDL-C and low density lipoprotein cholesterol (LDL-C) compared to the T2D participants.

**Table 1:** Basic characteristics of UCLEB participants with and without T2D

Trait	No-T2D participants		T2D participants		p value
	n	Trait	n	Trait	
Age (years)	13,015	61.1 (6.0)	1803	61.3 (8.1)	0.32
Sex (percentage male)	8068	62.00	1053	58.4 %	0.003
BMI (kg/m^2)^	12,803	26.7 (4.3)	1747	28.6 (5.80)	1.346 × 10^−36^
Triglycerides (mmol/l)^a^	12,022	0.43 (0.55)	1563	0.67 (0.75)	8.461 × 10^−33^
Total Cholesterol (mmol/l)	12,736	6.28 (1.24)	1784	6.04 (1.65)	4.484 × 10^−8^
HDL-cholesterol (mmol/l)	12,493	1.42 (0.38)	1757	1.25 (0.51)	2.114 × 10^−34^
LDL-cholesterol (mmol/l)	12,385	4.00 (1.07)	1607	3.62 (1.43)	1.573 × 10^−21^
Systolic blood pressure (mmHg)	12,739	139.90 (22.80)	1783	148.00 (30.60)	1.650 × 10^−23^
Diastolic blood pressure (mmHg)	12,722	81.70 (12.90)	1782	84.40 (17.30)	3.716 × 10^−9^
Fasting glucose (mmol/l)^a^	12,741	1.69 (0.15)	1670	1.98 (0.19)	2.54 × 10^−303^
Insulin (µIU/ml)^a^	7732	1.89 (0.62)	456	2.50 (0.66)	1.686 × 10^−80^
Glycated haemoglobin (%)	8711	5.37 (0.65)	1807	6.80 (0.98)	8.14 × 10^−265^

Mean and standard deviation of each trait (where applicable) is shown in those with and without T2D. Results were adjusted for age and sex

^a^Variables were log transformed

### Rs10911021 and CHD in T2D

**Fig. 1:**
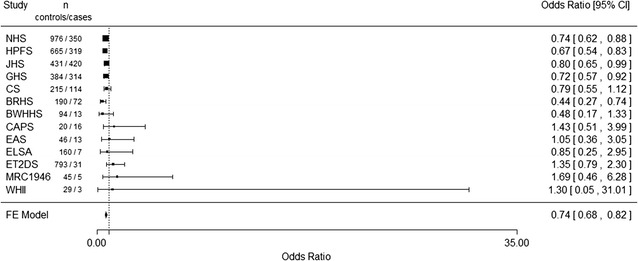
*Forest plot* of the meta-analysis (fixed effects) of UCLEB studies and published data for the relationship between rs10911021 and coronary heart disease in diabetic individuals

The SNP, rs10911021, had been imputed in eight of the UCLEB studies that also had data on diabetes status as shown in Table 2. The association between rs10911021 and CHD in diabetic participants was directionally similar to that previously reported but not statistically significant, OR 0.80 (95 % CIs 0.60–1.06, p = 0.13, MAF = 0.26) for the minor allele. The results from the UCLEB studies were meta-analysed with the published data. Similar effect sizes were observed using both fixed effects and random effects models with both p values strongly statistically significant, OR 0.74, 95 % CIs 0.68–0.82, p = 8.22 × 10^−10^ (Fig. 1) and OR 0.75 95 % CIs 0.67–0.84, p = 1.61 × 10^−6^, respectively. Heterogeneity between the studies was low (I^2^ = 18 %). No association between rs10911021 and CHD in those without T2D was observed, OR 1.00 (95 % CIs 0.92–1.10, MAF = 0.30) for the minor allele.

**Table 2:** Risk allele frequency of rs10911021 for UCLEB participants

	BRHS	BWHHS	CAPS	EAS	ELSA	ET2DS	MRC1946	WHII	Combined
No T2D									
MAF no CHD	0.30 (1544)	0.32 (1528)	0.31 (1022)	0.31 (553)	0.30 (1426)	–	0.32 (2294)	0.31 (2851)	0.31 (8665)
MAF CHD	0.30 (378)	0.31 (285)	0.28 (239)	0.29 (132)	0.29 (114)	–	0.31 (65)	0.35 (161)	0.30 (1677)
OR (95 % CI)	1.02 (0.85–1.22)	1.01 (0.79–1.28)	0.82 (0.65–1.04)	0.90 (0.67–1.23)	1.10 (0.81–1.49)	–	1.00 (0.68–1.47)	1.23 (0.97–1.56)	1.00 (0.92–1.10)
p value	0.81	0.94	0.10	0.64	0.54	–	0.43	0.09	0.93
T2D									
MAF no CHD	0.31 (190)	0.34 (94)	0.30 (20)	0.23 (46)	0.32 (160)	0.30 (793)	0.28 (45)	0.31 (29)	0.30 (1377)
MAF CHD	0.18 (72)	0.20 (13)	0.29 (16)	0.24 (13)	0.29 (7)	0.32 (31)	0.40 (5)	0.30 (3)	0.26 (160)
OR (95 % CI)	0.44 (0.26–0.74)	0.48 (0.17–1.33)	1.43 (0.51–4.00)	1.05 (0.36–3.03)	0.85 (0.25–2.94)	1.35 (0.80–2.33)	1.69 (0.46–6.25)	1.01 (0.52–1.96)	0.80 (0.60–1.06)
p value	2 × 10^−3^	0.16	0.49	0.95	0.80	0.26	0.43	0.87	0.13

Minor allele frequency *RAF* is shown separately for those who did and did not go on to develop CHD. n is shown in brackets. The odds ratio (OR) adjusted for sex for the association between rs10911021 and CHD in T2D is also shown with its 95 % confidence intervals (95 % CI)

### rs10911021 and the γ-glutamyl cycle in T2D

In order to investigate the relationship between rs10911021 and the γ-glutamyl cycle in T2D, we sought to determine whether this SNP was associated with levels of amino acids involved in the pathway. We analysed the relationship between rs10911021 and the levels of nine amino acids, which can be taken up into the cell via the γ-glutamyl pathway, determined using the NMR metabolomics platform. These included the metabolic intermediate glutamine and glutathione constituent glycine. No association between any of the amino acid measurements and rs10911021 in diabetic participants was found (Table 3). Similarly no association between the measures and rs10911021 was observed in those without T2D (data not shown).

**Table 3:** Relationship between rs10911021 and NMR-determined amino acid measures

Trait (mmol/l)	Beta-coefficient (se)	p value
Alanine	−0.007 (0.07)	0.94
Glutamine	0.005 (0.08)	0.94
Glycine	0.003 (0.07)	0.97
Histidine	0.03 (0.07)	0.66
Isoleucine	0.02 (0.07)	0.74
Leucine	−0.005 (0.07)	0.94
Valine	0.06 (0.07)	0.44
Phenylalanine	0.04 (0.07)	0.58
Tyrosine	−0.03 (0.07)	0.65

Beta-coefficient corresponding to the minor allele are shown, along with the standard error

### rs10911021 and conventional risk factors for CHD and T2D

We then sought to investigate if there was an association between rs10911021 and CRFs for CHD and T2D in UCLEB and whether this differed according to the presence of diabetes. As shown in Table 4, there was no association between any of the traits and rs10911021 for the non-T2D participants (p > 0.05), while in diabetic participants, the only significant association observed was with HDL-C levels (p = 0.0005). Surprisingly, given it had previously been reported as the CHD protective allele, the minor allele of rs10911021 was associated with 0.034 mmol/l lower HDL-C levels. The major allele appears to show a recessive effect as shown in Table 5.

**Table 4:** Relationship between rs10911021 and risk factors for CHD and T2D in UCLEB in participants with and without T2D

Trait	Number of non-T2D participants	Beta-coefficient in non-T2D participants (se)	p value	Number of T2D participants	Beta- coefficient in T2D participants (se)	p value
BMI (kg/m^2^)	12,803	−0.032 (0.055)	0.56	1747	−0.055 (0.178)	0.76
Triglycerides (mmol/l)^a^	12,022	0.007 (0.007)	0.34	1563	0.030 (0.020)	0.87
Total cholesterol (mmol/l)	12,736	−0.011 (0.016)	0.25	1784	0.026 (0.043)	0.54
HDL cholesterol (mmol/l)	12,493	−0.001 (0.005)	0.86	1757	−0.034 (0.012)	0.0005
LDL cholesterol (mmol/l)	12,385	−0.018 (0.014)	0.21	1607	0.070 (0.037)	0.06
Systolic blood pressure (mmHg)	12,739	0.045 (0.298)	0.88	1783	0.056 (0.794)	0.94
Diastolic blood pressure (mmHg)	12,722	0.052 (0.170)	0.76	1782	−0.510 (0.432)	0.24
Fasting glucose (mmol/l)^a^	12,740	0.001 (0.002)	0.61	1670	−0.011 (0.009)	0.21
Insulin (µIU/ml)^a^	7732	−0.019 (0.011)	0.09	456	0.039 (0.063)	0.53
Glycated haemoglobin (%)	8711	−0.003 (0.008)	0.73	1317	0.032 (0.040)	0.42

Beta-coefficient and standard error for each trait in those with and without T2D is shown. The beta effect relating to the minor allele is shown

^a^Variables were log transformed

**Table 5:** Mean HDL-C level by rs10911021 in UCLEB participants with and without T2D

	rs10911021 Genotype			p value
	CC	CT	TT	
T2D participants				
HDL-C (mmol/l)	1.35 (0.35)	1.31 (0.32)	1.31 (0.33)	0.0005
N	859	740	158	
No T2D participants				
HDL-C (mmol/l)	1.14 (0.35)	1.40 (0.36)	1.31 (0.33)	0.86
N	5975	5400	1118	

Mean and standard deviation are shown (adjusted for sex and study). C is the common, risk allele

**Fig. 2:**
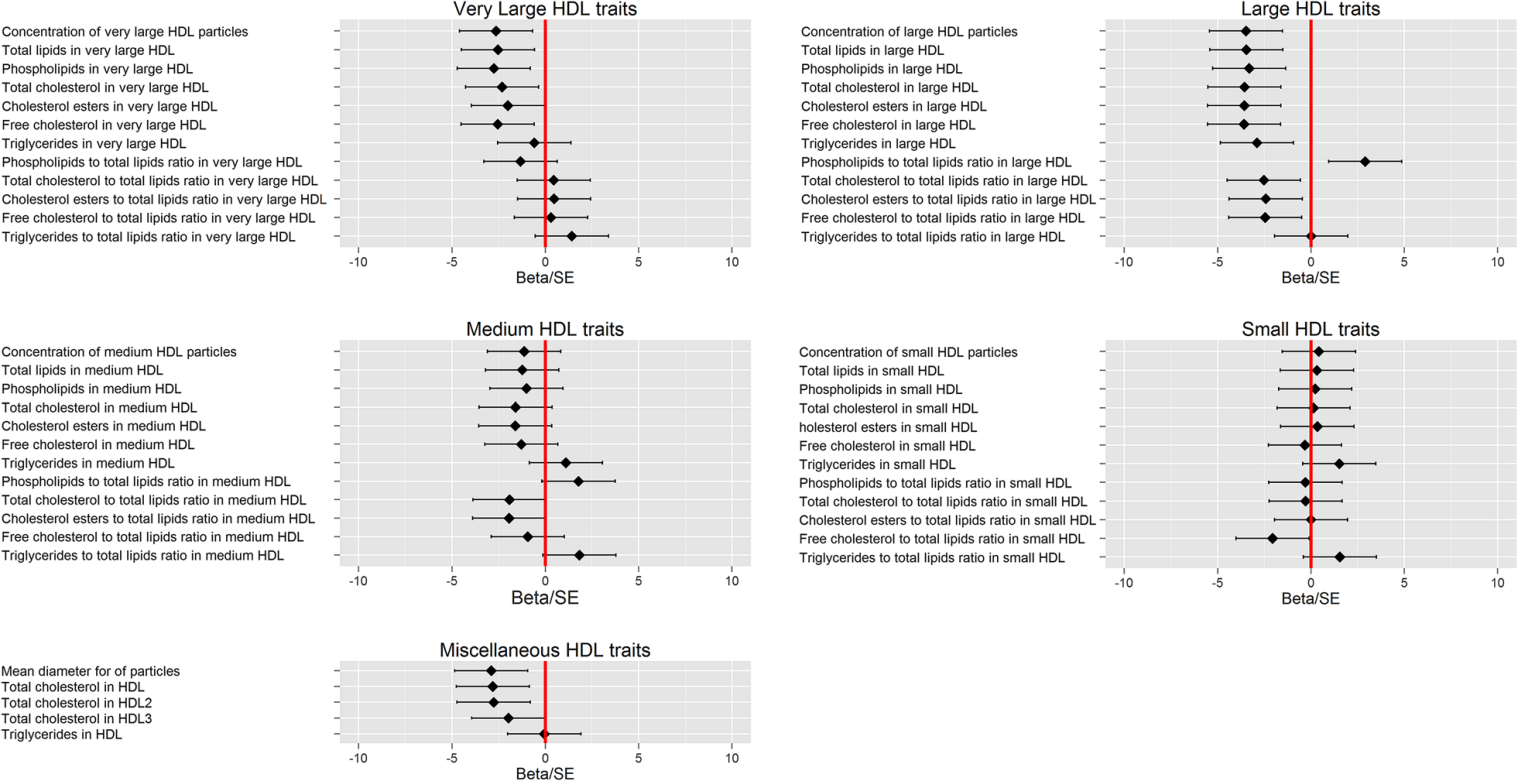
Relationship between HDL metabolomic traits and minor allele of rs10911021 in diabetic participants

**Fig. 3:**
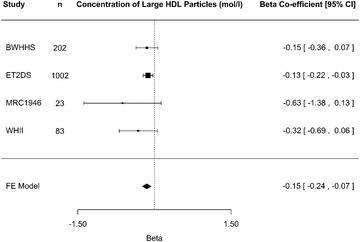
*Forest plot* for the meta-analysis of large HDL particle concentration and minor allele of rs10911021 diabetic participants

To further investigate the relationship between rs10911021, diabetes status and HDL, the association was examined with HDL traits measured by the NMR-metabolomics platform. Using this technique, HDL particles can be separated into four subclasses (very large, large, medium and small) with twelve lipid composition traits measured in each subclass. Overall mean HDL particle diameter, concentrations of HDL-C and the sub-fractions HDL2 and HDL3 and the triglyceride content of HDL particles were also measured. As shown in Table 6 in diabetic participants, six metabolic measures, all relating to large HDL particles, showed an association with rs10911021 with an FDR adjusted p value <0.05. A further 16 HDL metabolic measures had unadjusted p values below p = 0.05 (Fig. 2; Additional file 1: Table S3). By contrast, we found no association between rs10911021 and any of the HDL measurements in non-T2D participants (p > 0.05, Additional file 1: Table S4). Figure 3 is a representative forest plot of large HDL particle concentration showing a consistent lower level associated with the minor allele of rs10911021 diabetic participants in the four studies. Although metabolomics results are better interpreted as a profile rather than isolated associations, when the trait with the lowest p value (free cholesterol in large HDL) was included in the model for the five other statistically significant traits no other associations were observed (unadjusted p value >0.05, FDR p value = 1, Table 7). Therefore, we can conclude the associations observed reflect a common underlying effect.

**Table 6:** Metabolomic HDL traits with an association with rs10911021 in diabetic participants

Trait (mol/l)	Number of non-T2D participants	Beta-effect in non-T2D participants (se)	p value	FDR adjusted p value	Heterogeneity [I^2^ (%)]	Number of T2D participants	Beta-effect in T2D participants (se)	p value	FDR adjusted p value	Heterogeneity [I^2^ (%)]	p value for conditional analysis with rs1689800	FDR adjusted p value for conditional analysis with rs1689800
Concentration of large HDL particles	5221	0.01 (0.02)	0.59	1	0	1310	−0.15 (0.04)	0.0005	0.03	0	0.001	0.07
Total lipids in large HDL	5229	0.01 (0.02)	0.62	1	0	1310	−0.15 (0.04)	0.0005	0.03	0	0.001	0.07
Phospholipids in large HDL	5223	0.01 (0.02)	0.59	1	0	1310	−0.14 (0.04)	0.0009	0.03	0	0.002	0.09
Total cholesterol in large HDL	5223	0.008 (0.02)	0.71	1	0	1310	−0.15 (0.04)	0.0004	0.04	0	0.001	0.07
Cholesterol esters in large HDL	5221	0.009 (0.02)	0.67	1	0	1310	−0.15 (0.04)	0.0004	0.03	0	0.001	0.07
Free cholesterol in large HDL	5221	0.005 (0.02)	0.83	1	0	1310	−0.16 (0.04)	0.0003	0.03	0	0.0009	0.07

Beta-effects corresponding to the minor allele are shown, along with the standard errors. FDR analysis was performed using the Benjamini-Hochberg-Yekutieli method. When the use of lipid lowering therapy was not included in the linear regression model, the results remained directionally similar, but the statistical significance of the effects was reduced

*FDR* false discovery rate

**Table 7:** Metabolomic HDL traits with an association with rs10911021 in diabetic participants, adjusted for the trait with the lowest p value

Trait (mol/l)	Beta-effect (se)	p value	FDR adjusted p value
Concentration of large HDL particles	6.70 × 10^−4^ (0.01)	0.95	1
Total lipids in large HDL	5.28 × 10^−4^ (0.01)	0.96	1
Phospholipids in large HDL	4.38 × 10^−3^ (0.01)	0.74	1
Total cholesterol in large HDL	1.40 × 10^−3^ (0.01)	0.85	1
Cholesterol esters in large HDL	2.81 × 10^−3^ (0.01)	0.75	1

Beta-effects corresponding to the minor allele are shown, along with the standard errors. FDR analysis was performed using the Benjamini-Hochberg-Yekutieli method. The trait with the lowest p value (free cholesterol in large HDL) was included in the model for the five other traits found to be associated with rs10911021 in diabetic individuals. No other association were observed

*FDR* false discovery rate

Finally we sought to determine if the suggestive associations seen between rs10911021 and the large HDL particle measures were independent of the nearby GWAS HDL hit (lead SNP rs1689800). We performed a conditional analysis and found similar association between rs10911021 as in the unadjusted model (Table 6).

## Discussion

### The relationship between rs10911021 and CHD in T2D

In this study we observed a directionally consistent protective association between the minor allele of rs10911021 and CHD in diabetic participants and confirmed the lack of an association between the variant and CHD in those without T2D. Although the protective effect was more modest than previously reported and was not statistically significant in our sample, the power calculation showed that to have more than 80 % power to detect the effect found by Qi et al. (OR 0.74, for the minor allele), 237 CHD cases and 2038 CHD controls would be required. Here 160 cases and 1377 controls were analysed. Since the initial report of an association is often inflated by the “winners curse” effect, the lower risk estimate seen here is not unexpected. When the data presented here were meta-analysed with the previously published data using a fixed-effects meta-analysis, the p value was lower compared to the one found by Qi et al. indicating that our data support the original observation. A meta-analysis using a random effects model, to adjust for the observed heterogeneity between studies, was also performed although the p value was higher than in the original study. However, sensitivity analysis (Additional file 1: Tables S2, S3) shows that this is being driven by one study and as heterogeneity is relatively low between the studies, we are satisfied the fixed-effects model is suitable. In agreement with this, in the Look AHEAD cohort of overweight and obese individuals with T2D, rs10911021 was found to be associated with CVD [21]. The SNP was also found to be associated with all-cause mortality (and cardiovascular mortality) in diabetic individuals [22].

### The relationship between rs10911021 and the γ-glutamyl cycle

Qi et al. observed that subjects homozygous for the risk allele of rs10911021 had lower expression of the enzyme glutamine synthase (encoded by *GLUL)* in endothelial cells and a concomitant reduction in the ratio of pyroglutamic acid to glutamate, the substrate of the encoded glutamine synthase. The metabolomics platform data available did not directly measure either of these metabolites, but we did not find an association between the SNP and any of the amino acids measured. Included among these were glycine (one of the three constituents of glutathione along with cysteine and glutamate [23]) and glutamine which is the product of the reaction catalysed by the gene product of *GLUL*. We did not have measures of cysteine and glutamate which are crucial to glutathione levels nor the ratio of pyro-glutamic acid to glutamic acid which was found to be associated with rs10911021 by Qi et al. [6]. Thus we cannot discount that rs10911021 affects the γ-glutamyl cycle, but if so our results indicate that it is not through limiting the availability of glycine or by inhibiting general amino acid translocation into the cell.

### The relationship between rs10911021, HDL and T2D

There was no association between rs10911021 and any of the classical CHD risk factors in those without T2D, while in diabetic participants only an association with HDL-C was observed. This is contrary to what was observed in the Look AHEAD cohort, where rs10911021 was not associated with any CRFs [21]. More in-depth analysis using the metabolomics data found there was an association between the SNP and six large-HDL traits, again only in diabetic participants. There were also suggestive associations between the SNP and a further 16 HDL traits, mostly relating to large and very large HDL particles. These associations were found to be independent of the nearby HDL GWAS hit marked by rs1689800.

The relationship between HDL-C and CHD remains unclear. Mendelian randomisation studies have failed to find a causal relationship between genetically low HDL-C and CHD [24, 25] and HDL-C raising therapies have failed to improve cardiovascular outcome [26]. This has shifted the focus from HDL-C concentration towards HDL particle subclasses. Increased levels of small HDL particles have been associated with increased risk of CHD but the converse is true of large HDL particles [27–29]. In our analysis we found an association between the minor (previously identified as CHD “protective”) allele and *lower* levels of large HDL particle traits including concentration and cholesterol content, which is the opposite of what might have be expected for a protective gene variant. A variant with a similar phenotype (HDL-raising but also associated with CHD) was recently identified in the *SCARB1* gene [30] providing further evidence that high HDL-C is not protective and may in some circumstances promote atherosclerosis. There may also be other factors to consider. A study of Japanese individuals with T2D found an interaction between a variant in a different gene enzyme involved in the γ-glutamyl pathway (coding for the γ -glutamyl-transferase enzyme) and HDL-C on the arterial stiffness marker brachial-ankle pulse wave velocity) [31]. This suggests that the γ-glutamyl pathway may interact with HDL metabolism in the diabetic state.

It is also unclear why rs10911021 should be associated with HDL-C and HDL particle traits in T2D but not in the general population. Diabetic dyslipidemia is characterised by high triglyceride levels, a high concentration of small dense LDL particles and a low HDL-C concentration [32]. This reduction in HDL-C concentration is mostly driven by a potentially pro-atherogenic reduction in the presence of larger HDL particles [28]. However, it may be that presence of the minor allele of rs10911021 leads to changes in HDL metabolism altering the composition of large HDL particles (such as the reduction in cholesterol content observed here). This could result in the particles themselves having a less atherogenic lipid composition compared to carriers of the risk allele despite the reduction in overall large HDL particle concentration. Of course this pre-supposes that large HDL particles play a protective role and are not confounded by another causal factor.

## Limitations

There are several limitations to our study. We were unable to fully investigate the hypothesis concerning the association of rs10911021 with CHD in diabetic participants as we only had 60 % power to detect the effect size found by Qi et al. [6]. Data concerning the duration of diabetes and kidney complications, which can influence CHD risk in diabetic individuals, were not available and thus could not be taken account of in our analysis. Measures of metabolites directly involved in the reported γ-glutamyl (glutamate and pyroglutamic acid) association were not available and thus we could not fully investigate the hypothesis put forward by Qi et al. In the metabolomics analysis, one study, ET2DS, contributed the majority of participants with T2D. All suggestive associations were lost when this study was left out of the meta-analysis as power was greatly reduced. While the results were adjusted for use of any lipid-lowering medications, data on the specific medication used were not available for analyses in UCLEB and this may have led to residual confounding. It has long been known that the relationship between a particular lipid-lowering medication and HDL-C varies greatly. For example, rosuvastatin and simvastatin have been found to have a much greater HDL-cholesterol raising ability compared to atorvastatin [33]. It is unknown how lipid-lowering medications may affect the HDL sub-fractions measured here. A study investigating the impact of statin use on the HDL traits measured here found that the concentration of very large HDL particles to increase and small HDL particles to decrease while the concentration of large and medium HDL particles was largely unaffected [34] but did not assess individual statins. Due to the very high proportion of the T2D group that are on lipid-lowering medication we were unable to perform any meaningful analysis after exclusion of those on lipid-lowering medication.

## Conclusions

In summary, our results support an association between rs10911021 and CHD in diabetic participants. However, our results suggest that rs10911021 does not impact upon CHD risk by limiting the availability of the glutathione constituent glycine or by inhibiting general amino acid translocation into cells. However, we did observe an association between rs10911021 and classically measured HDL-C levels in T2D only. We also found an association between rs10911021 and a number of large HDL particle traits. Counterintuitively, the minor “protective” allele was associated with the atherogenic phenotype in both classically measured HDL and the metabolomics large HDL traits pointing to a potential novel mechanism through which HDL particles could promote atherosclerosis.

## Additional file

10.1186/s12933-016-0435-0 Sensitivity analysis for fixed-effects meta-analysis of association between rs10911021 and CHD in T2D. **Table S2.** Sensitivity analysis for random-effects meta-analysis of association between rs10911021 and CHD in T2D. **Table S3.** HDL traits with a suggestive association with rs10911021 in diabetic participants. **Table S4.** HDL traits which did not show an association with rs10911021 in those with or without T2D. **Figure S1.** Flow-chart showing number of UCLEB participants with and without prevalent T2D used in the different analyses.

## Appendix

The UCLEB Consortium is composed of the following individuals: Tina Shah^1^, Jorgen Engmann^1^, Chris Finan^1^, Amand Floriaan Schmidt^1^, Aroon D. Hingorani^1^, Caroline Dale^2^, Pimphen Charoen^2^, Antoinette Amuzu^2^, Ghazaleh Fatemifar^2^, Juan P. Casas^2^, Claudia Langenberg^2^, Jon White^3^, Vincent Plagnol^3^, Frank Dudbridge^4^, Meena Kumari ^5,6^, Mika Kivimaki^6^, Stela McLachlan^7^, Jacqueline Price^7^, Christine Power^8^, Elina Hypponen^8^, Andrew Wong^9^, Ken Ong^9,10,^ Rebecca Hardy^9^, Diana Kuh^9^, Nicholas Wareham^10^, Tom Gaunt^11^, Debbie A. Lawlor^11^, Fotios Drenos^12,13^ Jackie Cooper^12^, Philippa J. Talmud^12^, Steve E. Humphries^12^, Reecha Sofat^14^, Yoav Ben-Shlomo^15^, Peter Whincup^16^, Richard Morris^17^, Barbara Jefferis^18^, Goya Wanamethee^18^ and Claudia Langenberg^19^

^1^Institute of Cardiovascular Science, University College London, London, United Kingdom

^2^Farr Institute of Health Informatics, University College London, London, United Kingdom

^3^University College London Genetics Institute, Department of Genetics, Environment and Evolution, London, United Kingdom

^4^Department of Non-Communicable Disease Epidemiology, London School of Hygiene and Tropical Medicine, London, United Kingdom

^5^Institute for Social and Economic Research, University of Essex, Colchester, United Kingdom

^6^Department of Epidemiology & Public Health, UCL Institute of Epidemiology & Health Care, University College London, London, United Kingdom

^7^Centre for Population Health Sciences, University of Edinburgh, Edinburgh, United Kingdom

^8^MRC Centre of Epidemiology for Child Health, Department of Population Health Sciences, UCL Institute of Child Health, University College London, London, United Kingdom

^9^MRC Unit for Lifelong Health and Ageing, London, United Kingdom

^10^MRC Epidemiology Unit, Institute of Metabolic Science, Addenbrooke’s Hospital, Cambridge, United Kingdom

^11^MRC Centre for Causal Analyses in Translational Epidemiology, School of Social and Community Medicine, University of Bristol, Bristol, United Kingdom

^12^Centre for Cardiovascular Genetics, British Heart Foundation Laboratories, Institute of Cardiovascular Science, University College London, University Street, London, UK

^13^Computational Medicine, School of Social and Community Medicine, University of Bristol and Medical Research Council Integrative Epidemiology Unit at the University of Bristol, Bristol, United Kingdom

^14^Centre for Clinical Pharmacology, University College London, London, United Kingdom

^15^School of Social and Community Medicine, University of Bristol, Bristol, United Kingdom

^16^Population Health Research Institute, St George’s, University of London, London, United Kingdom

^17^School of Social and Community Medicine, University of Bristol, Bristol, United Kingdom

^18^Department of Primary Care & Population Health, UCL Institute of Epidemiology & Health Care, University College London, London, United Kingdom

^19^MRC Epidemiology Unit, University of Cambridge School of Clinical Medicine, Institute of Metabolic Science, Cambridge Biomedical Campus, Cambridge, United Kingdom

## Abbreviations

BRHS: British Regional Heart Study
BWHHS: British Women’s Heart and Health Study
CAPS: Caerphilly Prospective Study
CHD: coronary heart disease
CRF: conventional risk factor
EAS: Edinburgh Artery Study
ELSA: English Longitudinal Study of Aging
ET2DS: Edinburgh Type 2 Diabetes Study
FDR: false discovery rate
GWAS: Genome Wide Association Study
HDL-C: high density lipoprotein-cholesterol
LD: linkage disequilibrium
LDL-C: low density lipoprotein-cholesterol
MAF: minor allele frequency
MRC1946: MRC National Survey of Health and Development
NMR: nuclear magnetic resonance
OR: odds ratio
T2D: type 2 diabetes
UCLEB consortium: University College London-London School of Hygiene and Tropical Medicine-Edinburgh-Consortium
WHII: Whitehall II
