# Supplementary material for: Variant rs10911021 that associates with coronary heart disease in type 2 diabetes, is associated with lower concentrations of circulating HDL cholesterol and large HDL particles but not with amino acids
Source: Cardiovasc Diabetol. 2016 Aug 22;15(1):115. doi: 10.1186/s12933-016-0435-0 (PMC4994200; doi:10.1186/s12933-016-0435-0)
Supplement: Supplementary file 1 — 10.1186/s12933-016-0435-0 Sensitivity analysis for fixed-effects meta-analysis of association between rs10911021 and CHD in T2D. Table S2. Sensitivity analysis for random-effects meta-analysis of association between rs10911021 and CHD in T2D. Table S3. HDL traits with a suggestive association with rs10911021 in diabetic participants. Table S4. HDL traits which did not show an association with rs10911021 in those with or without T2D. Figure S1. Flow-chart showing number of UCLEB participants with and without prevalent T2D used in the different analyses. [file 12933_2016_435_MOESM1_ESM.docx]

**Supplementary tables:**

Table S1: Sensitivity analysis for fixed-effects meta-analysis of association between rs10911021 and CHD in T2D

| Study Source | Study Left Out | Estimate | p-value |
| --- | --- | --- | --- |
| Qi *et al* [[1](#_ENREF_1)] | NHS | 0.75 | 3.54x10^-7^ |
| Qi *et a*l [[1](#_ENREF_1)] | HPFS | 0.76 | 4.20x10^-7^ |
| Qi *et al* [[1](#_ENREF_1)] | JHS | 0.73 | 5.33x10^-9^ |
| Qi *et al* [[1](#_ENREF_1)] | GHS | 0.75 | 3.37x10^-8^ |
| Qi *et a*l [[1](#_ENREF_1)] | CS | 0.74 | 1.91x10^-9^ |
| UCLEB | BRHS | 0.76 | 1.43x10^-8^ |
| UCLEB | BWHHS | 0.75 | 1.58x10^-9^ |
| UCLEB | CAPS | 0.74 | 4.69x10^-10^ |
| UCLEB | EAS | 0.74 | 6.70x10^-10^ |
| UCLEB | ELSA | 0.74 | 8.28x10^-10^ |
| UCLEB | ET2DS | 0.73 | 1.22x10^-10^ |
| UCLEB | MRC1946 | 0.74 | 5.19x10^-10^ |
| UCLEB | WHII | 0.74 | 7.84x10^-10^ |

Odds ratio for the effect relating to the minor allele is shown along with the p-value.

Table S2: Sensitivity analysis for random-effects meta-analysis of association between rs10911021 and CHD in T2D

| Study Source | Study Left Out | Estimate | p-value | Heterogeneity (I^2^ (%)) |
| --- | --- | --- | --- | --- |
| Qi *et al* [[1](#_ENREF_1)] | NHS | 0.76 | 3.67x10^-4^ | 24.57 |
| Qi *et al* [[1](#_ENREF_1)] | HPFS | 0.77 | 1.39x10^-4^ | 17.83 |
| Qi *et al* [[1](#_ENREF_1)] | JHS | 0.74 | 3.48x10^-5^ | 21.88 |
| Qi *et al* [[1](#_ENREF_1)] | GHS | 0.76 | 1.51x10^-4^ | 24.39 |
| Qi *et al* [[1](#_ENREF_1)] | CS | 0.75 | 2.03x10^-5^ | 24.19 |
| UCLEB | BRHS | 0.76 | 1.43x10^-8^ | 0 |
| UCLEB | BWHHS | 0.76 | 5.08x10^-6^ | 20.79 |
| UCLEB | CAPS | 0.74 | 3.42x10^-7^ | 15.73 |
| UCLEB | EAS | 0.75 | 3.62x10^-6^ | 22.55 |
| UCLEB | ELSA | 0.75 | 7.29x10^-6^ | 24.47 |
| UCLEB | ET2DS | 0.73 | 1.22x10^-10^ | 0 |
| UCLEB | MRC1946 | 0.75 | 3.98x10^-7^ | 15.98 |
| UCLEB | WHII | 0.75 | 6.05x10^-6^ | 24.10 |

Odds ratio for the effect relating to the minor allele is shown along with the p-value.

Table S3: HDL traits with a suggestive association with rs10911021 in diabetic participants

| Trait | Number of non-T2D participants | Beta-effect in non-T2D participants  (se) | p-value | FDR adjusted  p-value | Heterogenity  (I2 (%) | Number of T2D participants | Beta-effect in T2D participants  (se) | p-value | FDR adjusted  p-value | Heterogenity  (I2 (%) | p-value for conditional analysis with rs1689800 | FDR-adjusted p-value for conditional analysis with rs1689800 |
| --- | --- | --- | --- | --- | --- | --- | --- | --- | --- | --- | --- | --- |
| Concentration of very large HDL particles (mol/l) | 5229 | 0.0002 (0.02) | 0.99 | 1 | 0 | 1310 | -0.11  (0.04) | 0.008 | 0.15 | 0 | 0.02 | 0.31 |
| Total lipids in very large HDL (mmol/l) | 5222 | 0.0002 (0.02) | 0.99 | 1 | 0 | 1310 | -0.11  (0.04) | 0.01 | 0.17 | 0 | 0.02 | 0.34 |
| Phospholipids in very large HDL (mmol/l) | 5222 | -0.006  (0.02) | 0.77 | 1 | 0 | 1310 | -0.12  (0.04) | 0.006 | 0.12 | 0 | 0.01 | 0.25 |
| Total cholesterol in very large HDL (mmol/l) | 5221 | 0.007  (0.02) | 0.76 | 1 | 0 | 1310 | -0.10  (0.04) | 0.02 | 0.26 | 0 | 0.03 | 0.43 |
| Cholesterol esters in very large HDL (mmol/l) | 5221 | 0.01  (0.02) | 0.63 | 1 | 0 | 1310 | -0.11  (0.04) | 0.04 | 0.52 | 0 | 0.02 | 0.76 |
| Free cholesterol in very large HDL (mmol/l) | 5221 | 0.005  (0.02) | 0.83 | 1 | 15.66 | 1310 | -0.11  (0.04) | 0.01 | 0.17 | 22.36 | 0.001 | 0.33 |
| Triglycerides in large HDL (mmol/l) | 5221 | 0.01  (0.02) | 0.63 | 1 | 0 | 1310 | -0.13  (0.04) | 0.003 | 0.10 | 0 | 0.008 | 0.25 |
| Phospholipids to total lipids ratio in large HDL (%) | 5142 | -0.001  (0.02) | 0.95 | 1 | 0 | 1056 | 0.14  (0.08) | 0.004 | 0.10 | 36.34 | 0.008 | 0.25 |
| Total cholesterol to total lipids ratio in large HDL (%) | 5140 | 0.0001 (0.02) | 0.995 | 1 | 0 | 1056 | -0.12  (0.04) | 0.01 | 0.17 | 10.86 | 0.03 | 0.38 |
| Cholesterol esters to total lipids ration in large HDL (%) | 5140 | 0.008  (0.02) | 0.71 | 1 | 0 | 1056 | -0.12  (0.04) | 0.02 | 0.21 | 0 | 0.04 | 0.46 |
| Free cholesterol to total lipids ratio in large HDL (%) | 5140 | -0.003  (0.02) | 0.96 | 1 | 0 | 1056 | -0.12  (0.04) | 0.01 | 0.20 | 6.64 | 0.03 | 0.38 |
| Free cholesterol to total lipids ratio in small HDL (%) | 5217 | 0.02  (0.02) | 0.37 | 1 | 0 | 1310 | -0.09  (0.04) | 0.04 | 0.48 | 0 | 0.08 | 0.84 |
| Mean diameter of HDL particles (nm) | 5227 | 0.006  (0.02) | 0.77 | 1 | 0 | 1310 | -0.12  (0.04) | 0.004 | 0.10 | 0 | 0.01 | 0.25 |
| Total cholesterol in HDL (mmol/l) | 5221 | 0.01  (0.02) | 0.52 | 1 | 0 | 1310 | -0.12  (0.04) | 0.005 | 0.12 | 0 | 0.01 | 0.25 |
| Total cholesterol in HDL2 (mmol/l) | 5227 | 0.01  (0.02) | 0.50 | 1 | 0 | 1310 | -0.15  (0.04) | 0.005 | 0.12 | 23.80 | 0.01 | 0.25 |
| Total cholesterol in HDL3 (mmol/l) | 5221 | -0.0009 (0.02) | 0.97 | 1 | 3.18 | 1310 | -0.09  (0.04) | 0.006 | 0.53 | 0 | 0.04 | 0.51 |

Beta-effects corresponding to the minor allele are shown, along with the standard errors. FDR=false discovery rate. FDR analysis was performed using the Benjamini-Hochberg-Yekutieli method.

**Table S4: HDL traits which did not show an association with rs10911021 in those with or without T2D**

| Trait | Number of non-T2D participants | Beta-effect in non-T2D participants  (se) | p-value | FDR adjusted p-value | Heterogeneity  (I^2^ (%) | Number of T2D participants | Beta-effect in T2D participants  (se) | p-value | FDR adjusted p-value | Heterogeneity  (I^2^ (%) | p-value for conditional analysis with rs1689800 | FDR adjusted p-value for conditional analysis with rs1689800 |
| --- | --- | --- | --- | --- | --- | --- | --- | --- | --- | --- | --- | --- |
| Triglycerides in very large HDL (mmol/l) | 5221 | -0.006 (0.02) | 0.77 | 1 | 0 | 1310 | -0.03 (0.04) | 0.56 | 1 | 0 | 0.69 | 1 |
| Concentration of medium HDL particles (mol/l) | 5229 | 0.03 (0.02) | 0.17 | 1 | 0 | 1310 | -0.05 (0.04) | 0.26 | 1 | 0 | 0.42 | 1 |
| Total lipids in medium HDL (mmol/l) | 5224 | 0.03 (0.02) | 0.16 | 1 | 0 | 1310 | -0.05 (0.04) | 0.21 | 1 | 0 | 0.36 | 1 |
| Phospholipids in medium HDL (mmol/l) | 5224 | 0.03 (0.02) | 0.17 | 1 | 0 | 1310 | -0.04 (0.04) | 0.31 | 1 | 0 | 0.49 | 1 |
| Total cholesterol in medium HDL (mmol/l) | 5222 | 0.03 (0.02) | 0.19 | 1 | 0 | 1310 | -0.07 (0.04) | 0.11 | 0.96 | 0.94 | 0.20 | 1 |
| Cholesterol esters in medium HDL (mmol/l) | 5224 | 0.03 (0.02) | 0.21 | 1 | 0 | 1310 | -0.07 (0.04) | 0.11 | 0.96 | 0 | 0.19 | 1 |
| Free cholesterol in medium HDL (mmol/l) | 5224 | 0.03 (0.02) | 0.17 | 1 | 0 | 1310 | -0.06 (0.04) | 0.20 | 1 | 0 | 0.31 | 1 |
| Triglycerides in medium HDL (mmol/l) | 5221 | 0.02 (0.02) | 0.38 | 1 | 0 | 1310 | 0.05 (0.04) | 0.27 | 1 | 0 | 0.21 | 1 |
| Concentration of small HDL particles (mol/l) | 5229 | 0.02 (0.02) | 0.29 | 1 | 0 | 1310 | 0.02 (0.04) | 0.67 | 1 | 0 | 0.62 | 1 |
| Total lipids in small HDL (mmol/l) | 5222 | 0.02 (0.02) | 0.38 | 1 | 0 | 1310 | 0.01 (0.04) | 0.75 | 1 | 0 | 0.71 | 1 |
| Phospholipids in small HDL (mmol/l) | 5222 | 0.03 (0.02) | 0.11 | 1 | 0 | 1310 | 0.01 (0.04) | 0.82 | 1 | 0 | 0.67 | 1 |
| Total cholesterol in small HDL (mmol/l) | 5221 | -0.004 (0.02) | 0.83 | 1 | 44.90 | 1310 | 0.01 (0.04) | 0.89 | 1 | 0 | 0.98 | 1 |
| Cholesterol esters in small HDL (mmol/l) | 5221 | -0.01 (0.02) | 0.58 | 1 | 59.27 | 1310 | 0.01 (0.04) | 0.73 | 1 | 0 | 0.91 | 1 |
| Free cholesterol in small HDL (mmol/l) | 5221 | 0.02 (0.02) | 0.43 | 1 | 0 | 1310 | -0.01 (0.04) | 0.74 | 1 | 0 | 0.85 | 1 |
| Triglycerides in very small HDL (mmol/l) | 5221 | -0.006 (0.02) | 0.78 | 1 | 0 | 1310 | 0.07 (0.04) | 0.13 | 1 | 0 | 0.17 | 1 |
| Phospholipids to total lipids ratio in very large HDL (%) | 5143 | 0.0004 (0.02) | 0.99 | 1 | 0 | 958 | -0.07 (0.05) | 0.18 | 1 | 16.56 | 0.31 | 1 |
| Total cholesterol to total lipids ratio in very large HDL (%) | 5142 | 0.008 (0.02) | 0.72 | 1 | 0 | 958 | 0.02 (0.05) | 0.66 | 1 | 0 | 0.90 |  |
| Cholesterol esters to total lipids ration in very large HDL (%) | 5142 | 0.0004 (0.02) | 0.98 | 1 | 0 | 958 | 0.02 (0.05) | 0.64 | 1 | 52.42 | 0.83 |  |
| Free cholesterol to total lipids ratio in very large HDL (%) | 5142 | 0.02 (0.02) | 0.41 | 1 | 0 | 958 | 0.02 (0.05) | 0.76 | 1 | 66.02 | 0.84 | 1 |
| Triglycerides to total lipids ratio in very large HDL (%) | 5142 | -0.01 (0.02) | 0.51 | 1 | 15.40 | 958 | 0.02 (0.05) | 0.16 | 1 | 0 | 0.13 | 1 |
| Triglycerides to total lipids ratio in large HDL (%) | 5140 | 0.003 (0.02) | 0.88 | 1 | 0 | 1056 | 0.0005 (0.05) | 0.99 | 1 | 8.14 | 0.89 | 1 |
| Phospholipids to total lipids ratio in medium HDL (%) | 5221 | -0.01 (0.02) | 0.50 | 1 | 53.87 | 1309 | 0.08 (0.04) | 0.08 | 0.70 | 0 | 0.12 | 1 |
| Total cholesterol to total lipids ratio in medium HDL (%) | 5218 | 0.01 (0.02) | 0.63 | 1 | 21.40 | 1309 | -0.08 (0.04) | 0.05 | 0.55 | 0 | 0.09 | 0.90 |
| Cholesterol esters to total lipids ration in medium HDL (%) | 5221 | 0.009 (0.02) | 0.69 | 1 | 24.66 | 1309 | -0.08 (0.04) | 0.05 | 0.55 | 0 | 0.08 | 0.84 |
| Free cholesterol to total lipids ratio in medium HDL (%) | 5221 | 0.01 (0.02) | 0.50 | 1 | 0 | 1309 | -0.04 (0.04) | 0.34 | 1 | 0 | 0.42 | 1 |
| Triglycerides to total lipids ratio in medium HDL (%) | 5218 | -0.0009 (0.02) | 0.97 | 1 | 0 | 1309 | 0.08 (0.04) | 0.07 | 0.66 | 0 | 0.10 | 0.94 |
| Phospholipids to total lipids ratio in small HDL (%) | 5218 | 0.04 (0.02) | 0.06 | 1 | 60.62 | 1310 | -0.0 (0.04) | 0.77 | 1 | 0 | 0.92 | 1 |
| Total cholesterol to total lipids ratio in small HDL (%) | 5217 | -0.03 (0.02) | 0.13 | 1 | 66.43 | 1310 | -0.01 (0.04) | 0.77 | 1 | 0 | 0.55 | 1 |
| Cholesterol esters to total lipids ration in small HDL (%) | 5217 | -0.03 (0.02) | 0.15 | 1 | 60.49 | 1310 | -0.00005 (0.04) | 0.9996 | 1 | 0 | 0.74 | 1 |
| Triglycerides to total lipids ratio in small HDL (%) | 5217 | -0.01 (0.02) | 0.56 | 1 | 6.76 | 1310 | 0.07 (0.04) | 0.12 | 1 | 3.23 | 0.18 | 1 |
| Triglycerides in HDL (mmol/l) | 5219 | 0.01 (0.02) | 0.60 | 1 | 0 | 1310 | -0.002 (0.04) | 0.96 | 1 | 0 | 0.94 | 1 |

Beta-effects corresponding to the minor allele are shown, along with the standard errors. FDR=false discovery rate. FDR analysis was performed using the Benjamini-Hochberg-Yekutieli method.

Figure Legends:

Figure 1: Forest plot of the meta-analysis (fixed effects) of UCLEB studies and published data for the relationship between rs10911021 and coronary heart disease in diabetic individuals

Figure 2: Relationship between HDL metabolomic traits and minor allele of rs10911021 in diabetic participants

Figure 3: Forest plot for the meta-analysis of large HDL particle concentration and minor allele of rs10911021 diabetic participants

Figure S1: Flow-chart showing number of UCLEB participants with and without prevalent T2D used in the different analyses
